# Evaluating the role of surgical sterilisation in canine rabies control: A systematic review of impact and outcomes

**DOI:** 10.1371/journal.pntd.0008497

**Published:** 2020-08-26

**Authors:** Abi Collinson, Malcolm Bennett, Marnie L. Brennan, Rachel S. Dean, Jenny Stavisky

**Affiliations:** 1 School of Veterinary Medicine and Science, University of Nottingham, Sutton Bonington, United Kingdom; 2 VetPartners, York, United Kingdom; Universidad Nacional Mayor de San Marcos, PERU

## Abstract

Current recommendations for the elimination of canine-mediated human rabies focus on mass dog vaccination as the most feasible and cost-effective strategy. However, attempts to control rabies are often combined with canine surgical sterilisation programmes. The added value of sterilisation is widely debated. A systematic review was undertaken to compare the outcomes and impact of vaccination and sterilisation programmes with vaccination only programmes. A systematic search of three electronic databases (CAB Abstracts, Medline and Global Health) and grey literature was performed. From 8696 abstracts found, 5554 unique studies were identified, and 16 studies met the inclusion criteria. Eight described vaccination only programmes and eight described vaccination and sterilisation programmes. Indicators of impact measured were dog bites and/or doses of post-exposure prophylaxis administered; numbers of dog and/or human rabies cases; dog population demographic changes; changes in health and welfare of dogs, and indicators related to human behaviour change. The studies were contextually very diverse, programmes being implemented were complex, and there was variation in measurement and reporting of key indicators. Therefore, it was difficult to compare the two types of intervention, and impossible to make an evaluation of the role of sterilisation, using this evidence. Given the large number of vaccination and sterilisation programmes conducted globally, the lack of studies available for review highlights a gap in data collection or reporting, essential for impact assessment. There are several knowledge gaps concerning the impact of the sterilisation component alone, as well as subsequent effects on rabies transmission and control. Prospective studies comparing the outcomes and impact of the two interventions would be required in order to establish any additional contribution of sterilisation, as well as the underlying mechanisms driving any changes. In the absence of such evidence, the priority for rabies control objectives should be implementation of mass vaccination, as currently recommended by the World Health Organisation.

## Author summary

Rabies is an important viral zoonosis that causes approximately 59,000 human deaths every year. The vast majority of cases result from a bite from an infected dog. Annual vaccination of at least 70% of the dog population is recommended for elimination of canine rabies. Canine surgical sterilisation programmes are sometimes conducted alongside rabies vaccination. However the contribution of sterilisation to rabies control, above that of the vaccination alone, is controversial. This systematic review compared the outcomes and impacts of vaccination and sterilisation programmes with vaccination only programmes. Sixteen studies met the study’s formal inclusion criteria, however we were unable to answer the question using this evidence and the role of sterilisation remains unclear. We identify gaps in knowledge regarding the implementation and impact of the sterilisation component as well as any resulting effects on rabies transmission and control. Prospective studies evaluating the two types of intervention would provide comparative evidence, which is needed to enable informed decisions to be made regarding whether sterilisation should be conducted for the purposes of canine rabies control.

## Introduction

Many high-income countries have eliminated canine-mediated rabies, usually through a combination of vaccination and stray dog control. However, in many low and middle income countries the disease remains endemic, causing an estimated 59,000 human deaths globally per year [1]. Large populations of free-roaming dogs in these countries pose a challenge for control strategies. They may be unowned, semi-owned, difficult to handle or with owners/care-givers who do not engage in, or have access to, control strategies. Mass dog vaccination is widely accepted as the most feasible and cost-effective strategy for eliminating dog to human rabies transmission [2–5]. This approach is sometimes aligned with surgical sterilisation programmes, which may have different, but synergistic aims, including free-roaming dog health and welfare improvement and population reduction or stabilisation. The World Health Organisation (WHO) suggests sterilisation as a supplementary measure to vaccination, only when high vaccination coverage has been achieved and additional or separate funds are available [6]. In India, the Animal Birth Control (Dogs) Rules, 2001 instruct that all street dogs are to be sterilised, rabies vaccinated and released and this has been the approach taken in many Indian cities [7]. Large scale catch- neuter-vaccinate-release (CNVR) programmes have also been implemented in Bangladesh [8] and Bhutan [9]

One of the arguments against the use of sterilisation is that reductions in dog population size or density are not necessary for rabies control [2,10,11]. However, sterilisation theoretically stabilises the population through reducing birth rates, increasing dogs’ longevity and maintaining a healthier population. High population turnover is a recognised barrier to vaccination success [2,12]. Reduced population turnover should help increase vaccination coverage and might allow for extended time between campaigns [13]. Modelling supports a role for population control through sterilisation, particularly if sterilised dogs are permanently and readily identifiable, so that only new dogs are vaccinated in each campaign [14].

Sterilisation can also lead to changes in human attitudes and behaviours towards dogs [15], for example increased perception of safety, and pride in ownership of sterilised and vaccinated dogs and improved care-giving behaviours [16]. This may in turn enhance community support for, and engagement with, rabies vaccination campaigns. However, human behaviour change may also be affected by dog demographic changes, for example human mediated movement of dogs may increase after sterilisation programmes if demand for dogs is still high but birth rates are reduced [13], which could be detrimental to rabies control.

Sterilisation is often criticised as resource and time expensive, and potentially detrimental to rabies control if resources are diverted away from vaccination [17,18]. Vaccination only campaigns may be required in addition if there is inadequate coverage [8,9]. As the sterilisation component takes longer both to implement and to produce an impact, it has been recommended that sterilisation be viewed as a separate undertaking to rabies control [19].

Furthermore, evidence supporting increased longevity in sterilised animals typically comes from pet populations in high income countries [20]; extrapolation of these data to free-roaming dogs may not be valid. Average life-expectancies for free-roaming dogs differ from pets, and vary worldwide from 1.1 to 5 years [12], but how sterilisation affects this lifespan has not been determined. There is some evidence that sterilised free-roaming dogs have higher body condition scores than entire animals, potentially indicating improved welfare [9,21]. Entire dogs in cities with sterilisation programmes have also been found to have higher body condition scores and a significantly lower prevalence of several diseases, when compared with a city with no programme [22], potentially indicating indirect benefits to the total dog population.

Studies investigating the effects of sterilisation on free-roaming dog behaviour such as aggression and roaming, both of which have the potential to affect rabies transmission, have produced conflicting results. Female spaying has been associated with decreased dog bites [23], whereas male castration was found to have no effect on aggression or roaming behaviour [24]. Sex differences also add extra complexity as entire females and sterilised males have found to have more contact with other free-roaming dogs than sterilised females and entire males [25].

Impact assessment of dog population management programmes (DPM) is not common [26] and estimating the contribution of any component, such as sterilisation, in combined interventions is challenging [27]. The additional impact that sterilisation makes over vaccination alone in the control of rabies has not been fully evaluated [4].

The aim of this systematic review was to assess the role of surgical sterilisation in canine rabies control by comparing the reported outcomes and impact of vaccination and sterilisation programmes (V-S) with those of vaccination only (V) approaches.

## Methods

### Search strategy

A search of CAB Abstracts (1910- present), Medline In-Process and Non-Indexed Citations and Ovid Medline (1946-present) and Global Health CABI was performed in August 2017 using the OVID interface to identify studies that measured impacts of canine rabies control programmes that conducted either canine surgical sterilisation and rabies vaccination or rabies vaccination only.

The searches used combined terms for dogs (dog, dogs, canine, canines, canis), rabies (rabies, rabid) and vaccination or sterilisation (vaccination, vaccine, vaccinate, immunisation/immunization, immunise/immunize, sterilis$, steriliz$, dog population management, animal birth control, neuter$, spay$, spey$, castrat$, ovariohysterectomy, gonadectomy).

Additional studies for inclusion were identified by hand searches of relevant articles, expert referral and a grey literature search of conference proceedings and funding body reports (S1 File contains the protocol with further details). Online proceedings of relevant conferences were searched using the pre-defined search terms. Any initiatives referenced in key papers were followed up, if not already included or excluded. A broad range of individuals/organisations (n = 23) who had worked in dog population management and/or rabies control internationally, either in academia, for a governmental or a non-governmental organisation, were approached via email to provide any unpublished reports or details of any programmes they knew of that might have relevant data. A reminder email was sent after four weeks to non-responders.

### Study selection and criteria

Search results were exported into EndNote (Thomson Reuters, Philadelphia, PA, USA) and screened by a single author (AC) to determine if they met pre-determined inclusion and exclusion criteria (Table 1). Duplicates were removed using the EndNote function (n = 3094) and then manually during the screening process for those not discovered by EndNote (n = 466). If more than one study described the same programme then either the study with the most comprehensive presentation was used, or both were included if different impacts were reported. A second author (RD) screened 10% of the studies (after duplicates removed) for concordance. Any studies queried for inclusion were discussed with JS and RD.

**Table 1:** Inclusion and exclusion criteria for study selection.

Inclusion	Exclusion
Canine rabies control programme using rabies vaccination only or rabies vaccination and surgical sterilisation	Not describing canine rabies control programme
Intervention uses either rabies vaccination only or vaccination and surgical sterilisation (may have additional components such as education, access to further veterinary care)	Intervention also uses another method for population control which has a direct effect on population size e.g. culling, relocation or confinement e.g. permanent sheltering, or other forms of fertility control e.g. immunocontraception
Measured one or more of the following impacts: number of dog bites, number of confirmed or suspected rabid dog bites, number of dog rabies cases, number of human rabies cases, dog population turnover, changes in health and welfare of dogs, changes in knowledge, attitudes and/or practices towards dogs and/or the intervention	No impacts measured
Population compared at baseline and after or throughout an intervention	Required impacts only measured once
Intervention details were either accessible in a peer-reviewed journal or able to be obtained in full from another source or from the authors	Full intervention details unable to be obtained or insufficient description of the intervention to understand how the program was implemented
Available in English language, or another language with translation available at the University of Nottingham	

### Data extraction and synthesis

Pre-determined qualitative and quantitative data were extracted from each included study using a standardised data extraction form designed for this study (S2 File). The main sections were study characteristics (e.g. country, setting, length of study, publication type), intervention details (including activities), outcomes (vaccination coverage and sterilisation coverage) and indicators of impact (as described in Table 1). Contextual factors and programme components that may have influenced the success, or otherwise, of the intervention were also identified and extracted.

### Quality assessment

A quality appraisal was completed for each study, using a modified version of the appraisal tool for cross-sectional studies (AXIS) [28]. This was adapted to include only relevant questions and was reduced from 20 questions to 16 (S3 File). Methods for obtaining dog population size estimates, vaccination and/or sterilisation coverage and impacts measured were also evaluated, including potential biases associated with these methods. Risk of bias in individual studies e.g. potential confounders, and across studies was examined.

### Reporting

This systematic review is reported according to the Preferred Reporting Items for Systematic Reviews and Meta-Analyses (PRISMA) guidelines [29]. The complete PRISMA checklist can be found in the supporting information (S4 File)

## Results

**Fig 1:**
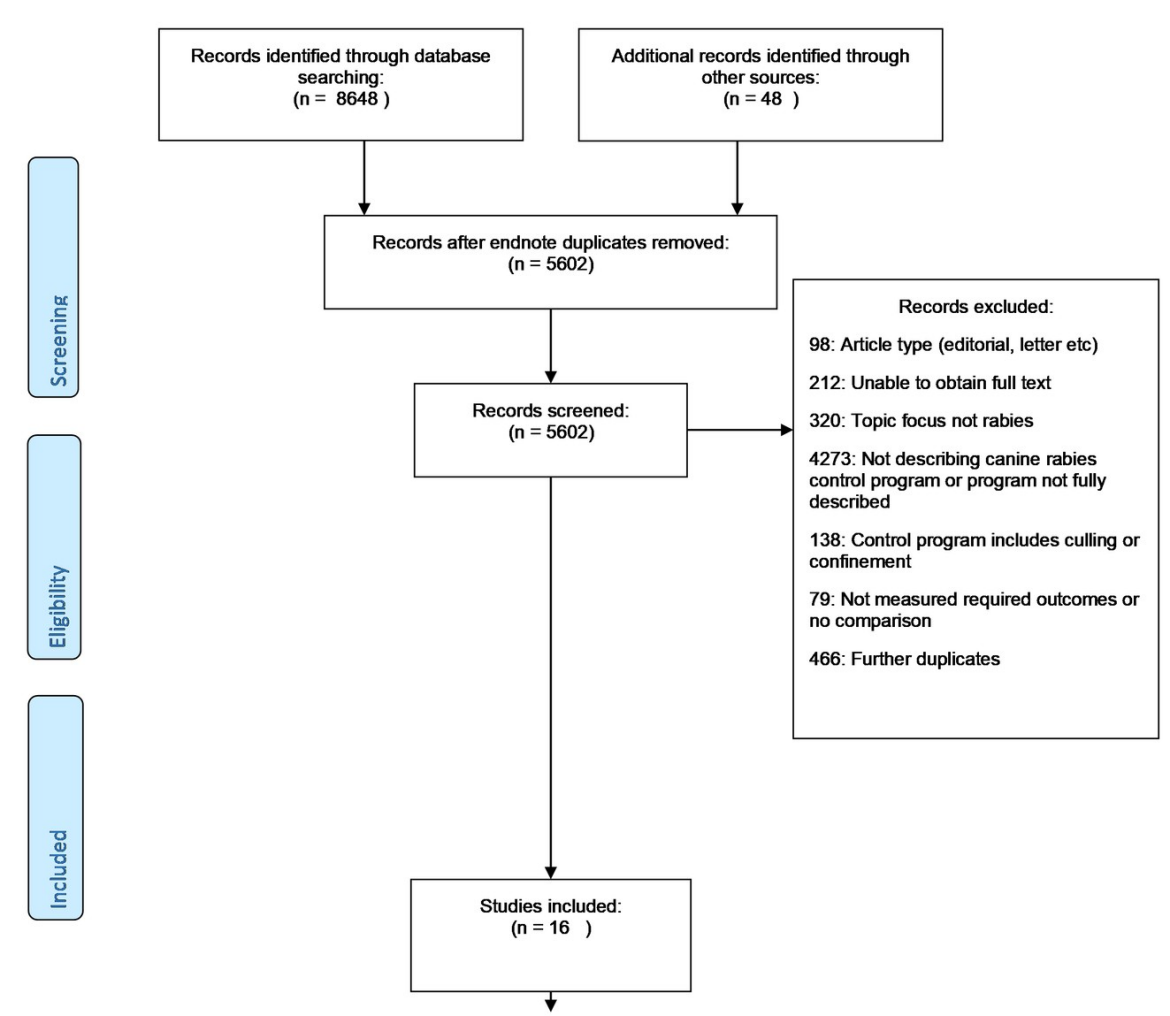
PRISMA flowchart. Selection of studies for inclusion.

The searches yielded 5554 unique studies from 8648 abstracts identified by the database searches (CAB = 3464; Medline = 2094; Global Health = 3090). A further 48 studies were identified in the grey literature. After abstract and full-text review, 16 studies were eligible for inclusion. Eight described V-S programmes [23,30–36] and eight V programmes [37–44] (Fig 1).

### Summary of included studies

Included studies were published between 1988 and 2018 and were predominantly peer-reviewed publications (n = 13); two reports and one conference proceeding were also included (Table 2). The eight V-S studies represented six different programmes. Different aspects of the same programmes in Jaipur [23,33] and Colombo [31,36] were reported in the included studies. All six V-S programmes were carried out in Asia, whereas the V programmes (n = 8) were conducted in Asia, Africa and South America. The length of study ranged from 1.5 to 10 years with a median of 4.5 years.

**Table 2:** Study characteristics.

Author and publication year	Location	Intervention area	Length of study (years)	Aims of study	Study design	Article type
**VACCINATION AND STERILISATION**						
Byrnes et al (2017) [30]	Sikkim, India	Province–predominantly rural (75%)	10	Perspective on implementation of the Sikkim Anti-Rabies and Animal Health (SARAH) program for the control and elimination of dog mediated human rabies in Sikkim	Observational, repeated cross sectional	Peer-reviewed
Hasler et al (2014) [31]	Colombo, Sri Lanka	Urban	4	Describe how different methods and data from multiple disciplines can be integrated in a One Health framework to provide decision-makers with relevant information, and apply it to a case study of rabies control	Observational, repeated cross sectional	Peer-reviewed
Kamoltham et al (2003) [32]	Phetchabun, Thailand	Rural	5	Results of rabies prevention programme implemented in Phetchabun province	Observational, repeated cross sectional	Peer-reviewed
Reece and Chawla [2006] (33)	Jaipur, India	Urban	8	Describe the effects of a rabies control program in a Northern Indian city	Observational, quasi-experimental, repeated cross sectional	Peer-reviewed
Reece et al (2013) [23]	Jaipur, India	Urban	8^a^ 13^b^	Determine if a relationship exists between canine reproductive behaviour and human dog-bites and if an ABC programme will reduce dog-bite frequency	Observational, repeated cross sectional	Peer-reviewed
Totton et al (2010) [34]	Jodhpur, India	Urban	2	Estimate age and gender demographics of the stray dog population in Jodhpur, proportion of stray dogs sterilised and vaccinated in the intervention and current impact of the program on stray dog population size.	Quasi-experimental, repeated cross-sectional	Peer-reviewed
Lee (2011) [35]	Koh Tao, Thailand	Island	2	Initiate, undertake and assess the effectiveness of a neutering and vaccination program on the welfare and number of dogs on the island	Observational, repeated cross-sectional	Report
WSPA (2010) [36]	Colombo, Sri Lanka	Urban	3	Initial stage of assessment to understand dog population dynamics, human-dog relationship and potential conflict (including risk of rabies) locally before developing a comprehensive program	Quasi-experimental, repeated cross-sectional	Report
**VACCINATION ONLY**						
Belotto (1988) [37]	Brazil	NR– 5 regions of country including large urban areas	5	Results of mass dog rabies vaccination campaigns in Brazil as a measure of reducing the incidence of rabies in urban areas of the country	Observational, repeated cross-sectional	Peer-reviewed
Chomel et al (1988) [38]	Lima-Callao, Peru	Urban	1.5	Results of a vaccination campaign conducted in Lima-Callao	Observational, repeated cross-sectional	Peer-reviewed
Cleaveland et al (2003) [39]	Serengeti District, Tanzania	Rural	4.5	Describe a vaccination strategy that has resulted in successful control of rabies in a rural dog population of Tanzania	Observational, Quasi-experimental, repeated cross-sectional	Peer-reviewed
Lechenne et al (2016) [40]	N’Djamena, Chad	Urban	2	Analysis of two consecutive dog mass vaccination campiagns conducted in N’Djamena to advocate the feasibility and effectiveness for rabies control through proof of concept	Observational, repeated cross-sectional	Peer-reviewed
Mpolya et al (2017) [41]	Southern Tanzania	Mixed—urban, rural and island	5	Experiences of implementing a large-scale rabies control project, a demonstration for the prevention of human rabies through the control and eventual elimination of canine rabies	Observational, Quasi-experimental, repeated cross-sectional	Peer-reviewed
Mudoga et al (2014) [44]	Zanzibar, Tanzania	Island	4	Description of a multisectorial approach to rabies control and elimination	Observational, repeated cross-sectional	Conference proceedings
Le Roux et al (2018) [42]	Kwa-Zulu-Natal, South Africa	Province—NR	7	Describe the KZN rabies project, established to eliminate human rabies through control of canine rabies and design a program that could be rolled out in neighbouring regions and countries.	Observational, repeated cross-sectional	Peer-reviewed
Valenzuela et al (2017) [43]	Ilocos Norte province, Philippines	Mixed–urban and rural	4	Test whether rabies could be eliminated in a province bordered by areas still endemic for rabies using a multi-sectoral model previously used in an island setting	Observational, Quasi- experimental, repeated cross-sectional	Peer-reviewed

^a^ Monthly human animal-bite data

^b^ Canine demographic and reproduction data

NR–not reported

Critical appraisal of the 16 studies identified that study design was not consistent and there were variations in outcomes and impacts measured and methods used, intervention details, study length and reporting. Study designs used could broadly be described as quasi-experimental, non-randomised, pre-post intervention or observational, repeated cross sectional. Impacts in the dog and/or human populations were measured or observed before and after an intervention had taken place, or during an ongoing intervention. Two studies [33,39] also compared an intervention area with a control area. Only four studies [23,31,34,39] determined the statistical significance of any of their findings.

### Intervention details

Another confounding factor affecting impact measurements both within and across studies was the concurrent use of other components (Table 3). Education and community awareness were common components of both types of intervention [30–32,35,40–43]. V-S programmes were more likely to have provision for free or subsidised veterinary care [30,31,33,35]. In comparison V programmes were more likely to improve access to post-exposure prophylaxis (PEP) for people [39,41–43].

**Table 3:** Comparison of intervention details.

Author and publication year	Programme (organisations involved in implementation)	Programme activities
**VACCINATION AND STERILISATION**		
Byrnes et al (2017) [30]	Sikkim Anti-Rabies and Animal Health (SARAH), (Government of Sikkim, Vets beyond Borders and Fondation Brigitte Bardot)	Sterilisation via fixed clinic and mobile units Vaccination initially house to house (HH) but as programme progressed central point (CP) became feasible in most villages. Treatment of sick and injured free-roaming dogs. Rabies prevention education.
Hasler et al (2014) [31], WSPA (2010) [36]	Colombo dog population management project (Colombo Municipal Council, Blue Paw Trust and World Society for the Protection of Animals (WSPA))	Sterilisation via mobile clinics and focus on female dogs. Vaccination of owned and unowned dogs. Basic veterinary treatment for low income communities. Education of children and adults in bite prevention and rabies awareness. Establishment of dog managed zones. Euthanasia of suspected rabid dogs.
Kamoltham et al (2003) [32]	Phetchabun province rabies control programme (Phetchabun Livestock Department and Public Health Office)	Mobile vaccination programme. Sterilisation targeting stray and community dogs. Increasing accessibility of PEP. Education of children and adults in rabies awareness
Reece and Chawla (2006) [33], Reece et al (2013) [23]	Help in Suffering	Fixed clinic. Catch-neuter-vaccinate-release—focus on sterilisation of females and prepubescent male dogs. Vaccination–at time of surgery for females and any dogs caught which had previously been sterilised were given a rabies booster. Humane euthanasia if necessary for health, welfare or behavioural issues
Totton et al (2010) [34] (with additional details of intervention from Totton et al (2011) [21]	Marwar Animal Protection Trust	Fixed clinic. Catch-neuter-vaccinate-release of free-roaming dogs >3 months old (dogs which were lactating or late stage pregnancy not captured)
Lee (2011) [35]	Noistar Thai Animal Rescue Foundation	Vaccination and sterilisation of owned, community and unowned dogs at fixed clinic and field sites. Simple veterinary services. Educational materials distributed via clinic.
**VACCINATION ONLY**		
Belotto (1988) [37]	(Public Health Services Foundation, Ministry of Health)	Vaccination implemented on one day in public places -distributed according to density of dog population and distance for owners. Mobile posts used in areas of low dog density.
Chomel et al (1988) [38]	(Ministry of Health, PAHO plus international organisations)	Vaccination implemented in one month–static point in accessible sites e.g. market places, public squares.
Cleaveland et al (2003) [39]	(Ministry of Water and Livestock Development)	Vaccination implemented on an approx. annual basis over 4.5y. Central point village based strategy. Aim of subsequent campaigns was to vaccinate previously unvaccinated dogs (e.g. pups born since previous campaign).
Lechenne et al (2016) [40]	(Institute de Recherche en Elevage pour le Developpement, Centre de Support en Sante, International and Swiss Tropical and Public Health Institute)	Vaccination implemented over 13 week campaigns in 2 consecutive years–Central point strategy (mobile on request and in the outskirts). Data used to get achieved coverage in real time and if below 70% and more dogs thought to be achievable then teams sent back to area.
Mpolya et al (2017) [41]	(WHO, government ministries from health and veterinary sectors, national and international research institutions)	Vaccination phased according to logistical constraints–started in urban areas and scaled up to include rural areas. Training local personnel. Community awareness programme. Decentalization of PEP provision.
Mudoga et al (2014) [44]	Zanzibar Rabies Prevention and Elimination Project (Ministry of Livestock and Fisheries, Ministry of Health and World Animal Protection)	Vaccination days in villages (sterilisation mentioned as being offered but no details given of this part of the intervention). Training local personnel and local village leaders. Education on responsible dog ownership.
Le Roux et al (2018) [42]	KwaZulu-Natal rabies project (Department of Environment, Agriculture and Rural Development, WHO and collaboration with animal welfare groups, academics, NGOs and human health sector)	Vaccination targeted using existing knowledge of local rabies epidemiology e.g. vaccinating dogs in potential source areas to stop transmission to adjacent areas–village based strategy. Education/awareness initiatives. Improve treatment for exposed people. Improve surveillance and diagnostics. Reported that initially used sterilisation but that this was slow and expensive with little overall impact on population size.
Valenzuela et al (2017) [43]	Ilocos Norte Communities against Rabies Exposure project (provincial rabies control committee, provincial veterinary and health offices and other local agencies)	Vaccination implemented annually, using fixed point and door-to-door strategy depending on geographic setting and preferences of the community.

### Outcomes

Table 4 shows a summary of reported programme outcomes and methods used for measurement (Table 4).

**Table 4:** Outcomes and methods for measurement.

Author and publication year	Vaccination coverage achieved^a^ (%)	Method for estimating vaccination coverage	Sterilis-ation coverage^b^ (%)	Method for estimating sterilisation coverage	Issues that may have impacted estimates
Byrnes et al (2017) [30]	18–85^b^	Vaccination numbers and dog population estimates	NR	n/a	Feral dogs in forests were excluded
Hasler et al (2014) [31]	NR	n/a	NR	n/a	
Kamoltham et al (2003) [32]	71^g^	NR–unclear if calculated from vaccination numbers or reported vaccinated by owner	NR	n/a	
Reece and Chawla [2006] (33)	35.5	Direct count during population surveys	65.7 females and 5.8 males	Direct count during population surveys	Vaccination coverage likely low estimate as based on ear-notched dogs (i.e. dogs that were also sterilised) only.
Reece et al (2013) [23]	NR	n/a	70–80 females	Direct counts along defined routes	NR
Totton et al (2010) [34]	61.8–86.5^b^	Proportion of notched dogs observed by marking team in each area	61.8–86.5^b^	Proportion of notched dogs observed by marking team in each area	Chained, leashed, confined, and/or collared dogs and puppies (<3m) were not included in population size counts
Lee (2011) [35]	89^c^	Household survey of dog owners	64^c^, >80 community/unowned dogs	Household survey for owned dogs, NR for community/unowned dogs	
WSPA (2010) [36]	80	NR	NR	n/a	
Belotto (1988) [37]	88.2	NR	n/a	n/a	
Chomel et al (1988) [38]	78^d^	Vaccination numbers and household survey	n/a	n/a	
Cleaveland et al (2003) [39]	67.8^f^	Household survey ^e^	n/a	n/a	
Lechenne et al (2016) [40]	71	Household survey and post vaccination transects. Bayesian statistical model	n/a	n/a	Vaccination coverage was the mean over all districts covered but coverage in each district varied
Mpolya et al (2017) [41]	65	Post-vaccination transects from 2013–15	n/a	n/a	Actual vaccination coverage likely lower as transects tended to miss young pups and campaigns not completed in every village
Mudoga et al (2014) [44]	70^c^	NR	NR	n/a	References a baseline dog population survey but NR in this study
Le Roux et al (2018) [42]	NR	Census in 2 villages but data NR	NR	n/a	
Valenzuela et al (2017) [43]	47.6	Dog censuses	n/a	n/a	Vaccination coverage data was not routinely collected as part of the vaccination campaign and therefore only indirectly assessed
	24.3	Household survey			
	13.1	Household surveys and dog counts corrected for incomplete detectability			

NR–not reported

n/a–not applicable

^a^ end of study NR–not reported

^b^ reported by region or area

^c^ owned dogs only

^d^ includes 13% vaccinated by private vets

^e^ average coverage over the 4 campaigns

^f^ coverage was also estimated for the first campaign using post vaccination transects and number of vaccine doses administered in relation to estimated dog population.

^g^ total over 6 campaigns

There were differences between studies in terms of methods and reporting of outcomes. Dog population size was often used for subsequent calculations of vaccination coverage. Methods for estimating population size used were extrapolations from human:dog ratios [39–41,43]; government censuses [30,32]; direct dog counts [33,36]; household surveys [35,43]; and mark-resight techniques [34,41]. Subsequent application of different calculations or models were sometimes used. Inaccurate dog population size estimates were acknowledged as a limitation in many of the studies, particularly when secondary sources of data e.g. government censuses or extrapolations using human:dog ratios were used. When multiple methods were used within the same study the wide variations in estimates that could be obtained were highlighted [40,41,43].

Vaccination coverage was reported in the majority of the studies [30,32–35,37–41,43,44]. Again, methods for estimation varied, or were not always reported [37,44]. The consequences of using unreliable dog population estimates to estimate vaccination coverage were demonstrated by Valenzuela et al [43], who found that coverage could be 13.1–47.6%, depending on the population estimation method used.

Vaccination coverage was also not reported in a consistent way across the studies. It varied between an average over the total intervention area, smaller geographical areas such as region, area or village or by differing sub-populations e.g. adults, sub-adults, owned, stray. Some studies included dogs vaccinated outside of the intervention in their final estimate, e.g. coverage estimated in a pre-intervention initial survey [40] or vaccinations performed at other places e.g. private vets [38]. With the exception of Cleaveland et al. [39] who attempted to vaccinate new dogs each year, and Reece et al. [23,33] who vaccinated when they sterilised, it was often unclear if it was largely the same or new dogs that were vaccinated each year. There were also differences in whether vaccination coverage was reported annually or just at the start and end of the study period.

Wide variations in vaccination coverage (13.1–89%) were associated with reductions in dog and/or human rabies, in both types of programme. One V-S programme reported a vaccination coverage of 35%, but discussed that this was likely to be a low estimate as it only included dogs that were also sterilised, whereas male dogs were vaccinated but not sterilised [33]. However interruption of rabies transmission was also reported in a V programme with coverage estimated between 13.1 and 47.6% [43]. This is not surprising as the critical coverage needed to interrupt rabies transmission is estimated to be 20–40%, the 70% recommendation is to take a high population turnover into account, and ensure that coverage doesn’t fall below this critical percentage [2].

Sterilisation coverage was less commonly measured, and was reported as proportions of different sub-populations, which hindered comparison. These were as a percentage of sterilised females (65.7%) and males (5.8%) in the free roaming dog population [33]; percentage of free-roaming dogs >3m sterilised in each study area (61.8–86.5%) [34] and as a proportion of owned (64%) or unowned (>85%) dogs [35]. Other studies often reported numbers of sterilisations conducted only [30,31,36].

### Impacts

Table 5 shows a summary of the results of the reported impacts measured in the included studies. Table 6 shows the methods used to measure each of these impacts.

**Table 5:** Summary results from included studies.

Author and publication year	Location	Reported Impacts
**VACCINATION AND STERILISATION**		
Byrnes et al (2017) [30]	Sikkim, India	• Initial decrease (2009–2010) then increasing trend (2010–2013) in dog-bites • Human rabies cases decreased from 4 (2006) to 0 (2007–2015) (BUT incursion in 2016–2 cases) • No consistent trend in size of dog population (increased, decreased and stayed the same in different areas)
Hasler et al (2014) [31], WSPA (2010) [36]	Colombo, Sri Lanka	• Decreased annual incidence of dog bites (0.0216–0.0143) in survey, increased number (131–160) reported at clinic • Dog rabies cases decreased (43–2) • No change in human rabies cases (3 in 4 years for pre and post intervention periods) • Dog population decreased after an initial increase (basic data NR) • % lactating females decreased (8% to 1.2%) • Decreased impact on animal welfare for intervention (compared to previous rabies control programme) • Increase in % dogs with good BCS and no visible skin conditions • Improved social acceptance scores between non-dog owners after programme • More problems reported concerning free-roaming dogs in the past in focus groups • Decrease in perception of rabies and breeding/puppies as problems • Difference in levels of roaming dogs reported in past and present
Kamoltham et al (2003) [32]	Phetchabun, Thailand	• Dog bites increased annually between 1997 and 2001 then decreased in 2001 • Human rabies cases decreased (3 to 0) • Dog population increased by 10%
Reece et al (2013 [23], Reece and Chawla (2006) [33]	Jaipur, India	• Decreased (4.91 bites per month) • Decreased human rabies cases (10 to 0) in intervention area, increased in non-intervention area of city • Dog population decreased (28%—average 3.5% per year) • No long term trend evident in proportion of females pregnant when sterilised
Totton et al (2010) [34]	Jodhpur, India	• Dog population size in 5 areas—decreased significantly in three areas, showed a non-significant decreasing trend in one area and did not change significantly in one area • Adults comprised majority of population at start and end of study. No clear pattern regarding higher prevalence of puppies or subadults
Lee (2011) [35]	Koh Tao, Thailand	• No change in dog bites (low overall) • Increase in dog population (700–903) • Decreased number dogs died due to disease/disappeared (28 to 15) • Increase in owned dogs and decrease in unowned and community dogs
**VACCINATION ONLY**		
Belotto (1988) [37]	Brazil	• Dog rabies cases decreased (4570 to 496–89% reduction) • Human cases decreased (168 to 52–69%)
Chomel et al (1988) [38]	Lima-Callao, Peru	• Dog rabies cases decreased, after May 1985 only 1 case (Dec 1985 –young pup not vaccinated in campaign) • Human cases decreased– 0 since campaign (baseline: 8, 5 (2y preceding campaign) and 3 in first ¼ year before campaign)
Cleaveland et al (2003) [39]	Serengeti District, Tanzania	• Significant decrease in annual incidence of dog bites (51%, 90% and 92% after each of 3 campaigns). In control zone incidence of bite injuries increased • Dog rabies cases significantly decreased (by 69.5–73.9%, 97.4–100% after 2 campiagns). In control zone no significant difference in incidence
Lechenne et al (2016) [40]	N’Djamena, Chad	• Dog rabies cases decreased (0.7/1000 to 0.073/1000)
Mpolya et al (2017) [41]	Southern Tanzania	• Increased number dog bites 2011–2012 then decreased but fluctuations later in study • Dog rabies cases in Pemba decreased (42 to 0 but recent incursion) Suspect cases. Major declines in South (data NR). Number submitted samples increased but proportion rabies positives decreased • Human cases decreased 17 to 0 in first 4y but then 4,4 and 2 in last 3y
Mudoga et al (2014) [44]	Zanzibar, Tanzania	• Decrease in dog bites (65%) • Dog rabies cases decreased (90%)
Le Roux et al (2018) [42]	KwaZulu- Natal, South Africa	• Dog rabies cases decreased (473 to 37) • Human cases decreased from mean 9.2 for pre-vaccination period to 0
Valenzuela et al (2017) [43]	Ilocos Norte province, Philippines	• Increasing trend in reported dog bites until final year of study • Decreased from 19–50 confirmed cases in pre-intervention years (average 35.5 cases and 38.8% +ve samples) to 0–8 confirmed cases (0–23% +ve) • Human cases decreased from suspect cases 2 to 0

NR–not reported

**Table 6:** Impacts measured and methods used.

Impact	Method for measurement	References using this method
**Dog bite incidents/PEP treatment given**	Department of Health records^a^	[30]
	Household survey and hospital records	[31]
	Hospital, clinic and health centre records^b^	[32]
	Hospital data	[23]
	Household survey	[35]
	Government District Hospital records^c^	[39]
	Ministry of Health records and mobile phone based surveillance	(41)
	Dog bite cases presenting to health facilities	(44)
	Animal Bite Treatment Centre records	(43)
**Dog rabies cases**	Veterinary department of Municipal Council records	[31]
	Not reported	[36,44]
	Ministry of Health records	[37,38]
	District Veterinary Office records and community based active surveillance measures	[39]
	Collected as part of routine diagnostic testing at research institute and based on dog population estimates	[40]
	Mobile phone based surveillance, contact tracing and samples submitted to labs	[41]
	Laboratory confirmed cases	[42]
	Regional animal disease diagnostic laboratory data—confirmed cases	[43]
**Human rabies cases**	Department of Health records (suspected cases)	[30]
	Municipal Council records	[31]
	Provincial Public Health report	[32]
	Infectious disease unit of main government hospital records	[33]
	Ministry of Health/Foundation SESP records	[37]
	Ministry of Health records	[38]
	Ministry of Health records and mobile phone based surveillance (suspected cases)	[41]
	Laboratory confirmed cases	[42]
	Department of Health records (suspected, probable or confirmed cases)	[43]
**Dog population size**	Estimate provided by village councils	[30]
	Census conducted by intervention	[32]
	Direct count (specified route)	[33]
	Mark recapture studies in 6 areas	[34]
	Direct count and household survey of dog owners	[35]
	Direct count (sample of wards)	[36]
**Dog population demographics**	Clinic records	[23]
	Direct count in specified areas	[34]
	Direct count (sample of wards)	[36]
**Health and welfare of free roaming dogs**	Qualitative scoring system for animal welfare assessment of intervened dogs	[31]
	No. dogs died due to disease/disappeared in last year–household survey	[35]
	Direct count	[36]
**Human behaviour changes**	Attitude statements (surveys) and focus groups	[31]
	Household survey	[35]

^a^ includes any potential rabies exposure

^b^ includes exposures to suspected and confirmed rabid animals

^c^ monthly per capita incidence calculated using 1988 human population government census data with projected growth rates

Dog rabies cases (n = 11) were most commonly reported, followed by dog-bite incidents (n = 9) and human rabies cases (n = 9). Least commonly reported impacts were dog population demographics (n = 3), dog health and welfare (n = 3) and indicators of human behaviour change e.g. public attitudes/perception (n = 2).

Impacts could not be compared between studies due to contextual differences in study area, study design and length, differences in methods of measurement and the presence of other components of the intervention. Even within studies, comparing impacts measured at the start and end did not necessarily allow for a comprehensive understanding of intervention effectiveness. Initial changes sometimes reversed over time [30,32,41], reflecting the dynamic nature of both dog populations and control programmes.

### Dog-bites, dog rabies and human rabies cases

There was conflicting evidence regarding the impact of vaccination with or without sterilisation on dog bite incidence, with increases (n = 2), decreases (n = 3) and no consistent trend (n = 4) all reported. Cleaveland et al. [39] found a significant decline in bites in a V programme. Reece et al. [23] proposed that sterilisation had an additional impact on dog-bite incidence as a result of its effect on dog bites that were due to maternal aggression. The different components of the interventions may address different motivations for dog bites. Reported changes also need to be evaluated in context, e.g. improved awareness may lead to increased reporting of dog-bites, rather than an actual increase in dog-bites. Hasler et al. [31] found a decreasing trend in dog bites in the household survey, whereas hospital records reported an increase, combining both sources of data demonstrated an actual increase in bite reporting. Focus groups and surveys suggested that this was due to better awareness of PEP and people being more likely to seek treatment.

Recorded numbers of dog and human rabies cases were often low in the included studies, even before the intervention. It was acknowledged in some papers that there were likely to be additional undiagnosed cases that were not reported [42], and references to weak surveillance systems [44]. Capacity for surveillance varied between studies, and this affected whether reported rabies cases were suspected or confirmed.

In at least one study [32] a change in the PEP programme was a large part of the intervention, so this was likely to have led to a reduction in human deaths, regardless of the canine component of the intervention. In another study [31] a well regulated PEP system was already in place before the intervention which meant the number of human rabies deaths was low even before the programme was implemented. This study saw no change in human rabies deaths. The remaining eight studies saw a decline in human rabies cases. Five reported a drop to zero cases by the end of the study [32,33,38,42,43]; a further two dropped to zero during the study but had cases towards the end [30,41].

Dog rabies cases showed a decline in all studies in which this outcome was measured. As with human cases, additional information such as surveillance capacity, is beneficial to assist in interpretation of these results as seen. For example, in Mpolya et al [41] a decrease in cases was reported, but also an increasing trend in samples submitted for testing. Activities to improve surveillance were not always noted in the publications, which is an important exclusion if efforts were made.

### Dog population demographics

Within the V-S studies there were conflicting results regarding effects on population size. However studies which reported a decrease used more robust methods for measurement [33,34,36]. Only V-S studies made repeated measurements of population size and were using it as an indicator of impact. The V studies that measured population size were doing so to assist in planning the intervention or for calculating vaccination coverage, and so only did so at one point within the study, or used different methods at different time points in the study.

Dog demography results from V-S studies reported no evident trend in proportion of females pregnant when sterilised (n = 1) [23] or age structure of population (n = 1) [34]. The percentage of lactating females decreased in the one study in which this was measured [36]. Dog health and welfare changes were not commonly measured. Hasler et al. [31] reported a higher welfare for dogs during the intervention, measured through a welfare assessment (n = 1), however this was in comparison to an intervention using culling rather than vaccination only, and was not looking at long term free-roaming dog welfare after the intervention. Other impacts measured were fewer dogs that had died due to disease or disappeared during the last year (n = 1) [35], and significantly higher body condition score plus absence of a visible skin condition (n = 1) [36]. Impacts related to dog population demographics, or the health and welfare of free roaming dogs were not reported in any of the V programmes.

### Human behaviour change

Indicators of human behaviour change were not commonly measured (n = 2) and measures used were not consistent between the studies. Impacts reported were a positive difference in social acceptance scores between non-dog owners at the start and end of the study, a decrease in the perception of dog related problems [31], and a large shift towards ownership, with a concurrent reduction in unowned and community dogs [35].

## Discussion

This systematic review compares the outcomes and impact of canine rabies control programmes using vaccination only or vaccination and sterilisation. Few publications made repeated measurements of the impacts required. Studies meeting selection criteria and included in the final analysis were very diverse in terms of primary aims, study design, length, intervention context and details, and data collection and analysis. It was not possible to assess the role of sterilisation in enhancing, or otherwise, vaccination as a means of controlling canine rabies using this evidence. The lack of studies available for review suggests that many V-S programmes are either not collecting or not reporting the data required for monitoring and evaluation of impact. These findings are similar to previous reviews of impact assessment in all DPM interventions, and DPM interventions involved in rabies control [26,27]. This is important because many organisations conduct sterilisation as part of their rabies control strategy. Several knowledge gaps were identified in relation to if and how V-S programmes affect the related impacts e.g. dog population turnover, as well as subsequent effects of these impacts on rabies transmission and control.

### Role of sterilisation

Although the included studies demonstrate examples of successful V-S programmes, evidence was not available to support an additional impact of the sterilisation component on dog or human rabies cases, time taken to see a reduction in these, or sustainability of results. For the related impacts associated with the theoretical benefits, there was generally evidence of positive effects on dog bites [23], population reduction [33,34], population turnover [35,36], increased health and welfare [36], and perception of and behaviour towards free-roaming dogs [31,35].

The V-S approach is often used in an attempt to achieve multiple objectives linked to managing free-roaming dog populations. These may include reducing human-dog conflict and improving the lives of free-roaming dogs. However, it is important to make a distinction between conducting sterilisation in order to achieve these wider objectives, and sterilisation for the purposes of rabies control. The effectiveness of V only programmes in reducing dog and human rabies cases has been demonstrated, both in studies included in this review, as well as additional evidence from various settings [2,45–48]. Therefore, programmes with rabies control objectives should focus on ensuring adequate vaccination, and consider sterilisation as a separate activity, with its own impacts to be assessed. This may help clarify any perceptions about a necessity for sterilisation in situations where rabies control is the priority.

Furthermore, the impact of sterilisation and vaccination on the related impacts is unclear and needs further investigation. Impacts on dog population demographics, dog health and welfare, and human behaviour change were less commonly measured than those measuring changes in dog-bites and dog and human rabies cases, and only in the V-S studies. This is unsurprising given that rabies control was an objective in all the papers, whereas V-S programmes were more likely to have additional objectives as discussed above. A better understanding of how vaccination alone affects these related impacts would be beneficial, both for evaluating any additional effects of sterilisation, and to provide additional insights for planning and implementing V programmes.

Dog population demography is an important factor in rabies control in terms of achieving and maintaining vaccination coverage. Evidence of how dog population demography may change in response to vaccination was not available in the included studies. Studies that have been conducted in relation to rabies vaccination tend to be cross sectional and are often conducted prior to an intervention to help inform planning [49,50]. Longitudinal studies are less common but can provide important insights into demographic changes occurring over time and factors that may regulate these changes [13,51]. A clear effect of vaccination on dog population demographics has not been found, although in one study from Tanzania, within vaccination villages survival was higher in vaccinated dogs than in unvaccinated dogs [52].

Human behaviour change is also important as human mediated movement of dogs is often identified as a source of rabies outbreaks [11,53,54] and participation in interventions is often the key to the success of rabies control programmes [55–57]. Positive changes in perception and ownership of free-roaming dogs after V-S programmes were reported [31,35]. However, human behaviour change is complex, and may be less to do with the sterilisation programme itself and more to do with factors such as presence in the community or trust in the organisation. In the Kwa Zulu-Natal programme, included as a vaccination only study [42], it was reported that sterilisation had initially been used but was discontinued due to expense and a lack of results seen in dog population demographics (data not reported). However a subsequent positive change in community attitudes towards veterinary services was attributed to their presence in communities for the sterilisation project, and led to an increase in vaccination numbers [58].

It was not possible to make an assessment of the effects of sterilisation on sustainability of impacts achieved by rabies control interventions. Included studies varied in duration, follow up, and time-point within the programme for which data was recorded. Within both V and V-S interventions, incursions of rabies were sometimes seen in later years [30,41]. Further studies have also described rabies outbreaks in areas that had previously seen interruption of transmission [40,54]. This is also linked to implementation and ability to scale up an intervention. V-S interventions are likely to be conducted on relatively small spatial scales due to longer implementation time, and expanding them geographically is likely to be challenging [59]. Geographical co-ordination is key as reintroduction of rabies from non-vaccinated areas is a challenge in rabies control [60].

Sustainability is also linked to economics, however a comparison of cost-effectiveness was outside the scope of this review. Mass vaccination has been demonstrated to be a cost-effective intervention [61], and sterilisation is far more resource intensive. Any benefits of sterilisation would have to be sufficient to justify the considerable additional costs. This has not been supported from modelling of the two strategies [14].

### Limitations

There are challenges with using systematic review methodology to evaluate complex interventions such as canine rabies control programmes. Difficulties in identifying and synthesising all relevant data are due to the lack of a standardised definition of the intervention, use of defined exclusion and inclusion criteria in study selection [62], and the importance of context and implementation on intervention impact [63].

The review demonstrates a large amount of variation in the implementation of the sterilisation component of V-S interventions. For example, if sterilisation of owned dogs was optional and reliant on owners or caregivers bringing dogs to a site [30] or if accessible dogs were caught and released [33,34]; or if there was a focus on females (and prepubescent males in Jaipur) [31,33]. Whilst these details may vary due to contextual factors and local adaptation may be needed, they are important for enabling an understanding of how impacts are achieved. There was also variation in reporting of the sterilisation component. In a number of the included studies, and outside of peer-reviewed publications, numbers of dogs sterilised is often the only measure of intervention effort and used as a representation for success. This may be due to widely held assumptions that intervention effort (i.e. number of dogs sterilised) is matched by effectiveness.

Improved characterisation of the sterilisation component including reporting of sterilisation coverage in terms of sex-specific proportions of defined sub-populations of dogs (i.e. owned, unowned, free-roaming or confined) would be beneficial in future research to allow an understanding of how impacts may be achieved. In contrast, whilst vaccination programmes may also vary in intervention details e.g. fixed point or house-to-house, they all have a common aim of achieving over 70% rabies vaccination coverage annually. The 70% target is sometimes applied to sterilisation too but there is no basis for this [27]. It may come from catch-neuter-vaccinate-release (CNVR) programmes in which vaccination and sterilisation coverage are the same.

Differences in contextual factors hindered meaningful comparison between the included studies. Geographical factors, local dog population dynamics, attitudes towards the dog population and baseline rabies prevalence are all likely to pose different challenges and affect the impact of control programmes. This is a challenge in all public health interventions, and establishing which components associated with success are a direct result of the intervention, and which are due to the context is important [64]. Different contexts may have different mechanisms at work in terms of how impacts are achieved. Identification of these mechanisms in future studies e.g. if sterilisation leads to a reduction of dog population turnover or reduction in abandonment of dogs, would enable a more comprehensive understanding of not just if sterilisation contributes to rabies control, but also why.

The perception of a programme in terms of ‘success’ may also vary across different locations and cultures, and is, to some extent, dependent on what the perceived problems were with free-roaming dogs at the outset of the intervention. In some settings, rabies control is not the priority for communities or policymakers with regard to free-roaming dogs. In a recent study from Chennai, only 15% of people interviewed cited rabies as a concern regarding free-roaming dogs [65]. In such settings there may not be a desire for vaccination-only campaigns, and the need to understand the benefit of sterilisation for DPM is more relevant. Sterilisation may also be able to act as an entry point for vaccination programmes in these settings [17].

In addition to variability in implementation of the sterilisation component, there were variations in what, if any, methods of population or rabies control had been used prior to the study period, as well as other components used during the study period. Interventions in which additional methods of population control, e.g. culling, were reported were excluded. However, additional components such as education initiatives, community awareness programmes and access to other veterinary care were present in the majority of included studies and had the potential to affect the outcomes measured. This highlights a major limitation with this study, which is that it is an oversimplification to classify interventions as V-S or V only. As well as the presence of other components, two of the studies, which were included as ‘vaccination only’, did refer to sterilisation programmes conducted in the study areas previously [44], or at some point in the intervention [42]. Furthermore, even if interventions were only using vaccination and sterilisation, it is difficult to separate out the specific effects of each component.

Few of the V-S studies used study designs that would allow causation to be determined, or attempted to partition the effects of the sterilisation component on rabies control impacts. This is likely due to many of the studies describing an ongoing programme, rather than being designed to answer a specific research question. In addition, variation in indicators measured, and methods used for measurement, made it difficult to make comparisons between studies regarding reported effects. An estimation of dog population size in the intervention area is particularly important. A range of different methods were used in the included studies, many of which have previously been found to have low validity [66]. The use of standardised indicators for relevant impacts would aid in synthesising evidence across interventions and settings.

Searches were carried out in English, and some studies may have been missed because of this, although several non-English language publications were included in the search results as they had English abstracts. The databases used for the searches were chosen because CAB Abstracts has been shown to have the widest coverage of veterinary literature and Medline is recommended if there is a biomedical aspect to the question [67]. Global Health is the only bibliographic database dedicated to Public Health, and benefits from good coverage of international literature.

Attempts were made to minimise publication bias by including grey literature in the review. We anticipated that many organisations may not necessarily be publishing their data. Despite the large number of organisations working in this field, few data were obtained by this route. Additional studies that were identified often either did not have sufficient methodological detail or did not include repeated measures of impacts. Reports from organisations often measured intervention effort only (i.e. vaccination or sterilisation numbers) or data that were collected remained unanalysed.

### Further work

In order to further investigate the question under review, prospective studies which compare the outcomes and impacts used in this review in an area, or comparable areas, using the two types of intervention would be needed. In V-S programmes, attempts should be made to partition the objectives and impacts of the different components, and the mechanisms believed to be at work should be identified. This will encourage deeper thinking about how best to implement each component and how to measure its impact. A clear description of contextual factors and intervention details is also important, as understanding the interaction between all of these factors is key in evaluating complex interventions [68].

### Conclusion

It is not possible to assess the impact of sterilisation, in addition to vaccination, to control canine-mediated rabies, based on the literature found in this systematic review. Prospective studies comparing outcomes and impact of the two types of intervention are needed if comparative evidence of effectiveness is to be obtained. In the absence of such evidence, a distinction should be made between the use of sterilisation for wider dog population management objectives rather than for rabies control objectives. There are many other social and ecological arguments for the use of sterilisation, and the impacts of sterilisation programmes warrants further investigation for these reasons. However, from the perspective of rabies control, unless evidence emerges that demonstrates an additional impact of sterilisation, the priority should be on implementation of mass vaccination.

## Supporting information

S1 File Study protocol. (DOCX)

S2 File Example data extraction form. (DOCX)

S3 File Modified AXIS checklist. (DOCX)

S4 File PRISMA checklist. (DOC)
