# Supplementary material for: Evaluating the role of surgical sterilisation in canine rabies control: A systematic review of impact and outcomes
Source: PLoS Negl Trop Dis. 2020 Aug 26;14(8):e0008497. doi: 10.1371/journal.pntd.0008497 (PMC7449413; doi:10.1371/journal.pntd.0008497)
Supplement: S1 File — (DOCX) [file pntd.0008497.s001.docx]

# S1: Systematic Review Protocol

**In canine rabies control programmes does sterilisation and vaccination combined compared with vaccination only lead to an improvement in outcomes?**

**Background**

Rabies remains endemic in many countries despite implementation of various rabies control programs. (WHO, 2013). Currently the most common aim for these programs is vaccination of at least 70% of the canine population. Dog population management (DPM) methods such as culling and sterilisation have also often been used in rabies control programmes.

It has been suggested that reducing dog population density is not effective for rabies control, due to the fact that rabies transmission is not thought to be density dependent. In fact there does not appear to be conclusive evidence to support either frequency-dependence or density-dependence in rabies transmission (Morters et al, 2013). In areas where culling has been carried out, repopulation will occur both due to new dogs moving into the area and people bringing in new dogs. Also vaccinated dogs may be included in the culling and new dogs are more likely to be susceptible meaning that herd immunity is reduced. However the impact of sterilisation on rabies transmission is less clear, and many interventions still perform sterilisation when working in rabies endemic areas. It is possible that other benefits provided by sterilisation such as reduced dog population turnover, lack of incoming dogs and changes in human behaviour may contribute to improved rabies control in other ways. Furthermore, evidence from some of these interventions have shown an improvement in indicators for reduction of rabies risk with a much lower rate of vaccination when carried out in conjunction with sterilisation (Reece and Chawla, 2006).

Local dog population ecology, the relationships between humans and dogs and human behaviour are important in the success of rabies control programs. (Beran and Frith, 1988). This is because the efficacy of a rabies vaccination program is dependent on factors such as the vaccinated dogs living long enough for herd immunity to increase. Populations with high numbers of stray dogs have been shown to have a high turnover, therefore potentially decreasing the impact of the vaccination effort. Another factor is the relationship between humans and dogs – do they bring their dog/dogs to be vaccinated and/or sterilised? If so, which ones and why? Are they confined or free roaming? Do they seek treatment for other diseases or health issues for the dogs?

This review aims to assess the contribution of sterilisation to rabies control by examining the current evidence base for canine rabies control programs and comparing the outcomes in programs where vaccination only has been carried out to those performing vaccination and sterilisation.

**Aims**

The aim of this review is to examine the role of surgical sterilisation in canine rabies control programs in terms of reduction of rabies risk. A secondary objective is to examine the role of sterilisation on other related outcomes such as dog population turnover, number of human dog bites, health and welfare of free roaming dogs and attitudes of the community to dogs, rabies control, sterilisation and the intervention.

**Objectives**

1. Identify the published, peer-reviewed literature on canine rabies control programs involving sterilisation and/or rabies vaccination.
2. Identify the grey literature on canine rabies control programs involving sterilisation and/or rabies vaccination.
3. Identify what outcomes of reduction of rabies risk and related outcomes have been measured in these studies and how success has been measured
4. Compare the success of sterilisation and vaccination programs versus vaccination only programs in terms of the greatest reduction of rabies risk, using these outcomes
5. Critically appraise the quality of the evidence base

**Search Strategy**

Databases

- Medline In-Process & Non-Indexed Citations and Ovid MEDLINE: 1946 - Present
- CAB Abstracts (Ovid): 1910 – present
- Global Health (Ovid): 1973 – present

Grey literature

- Dog population management conferences 2012 and 2015
- One Health EcoHealth conference 2016
- EcoHealth conferences (?2006, 2008), 2010, 2012, 2014
- One Health congress 2011, 2012, 2015
- GRF One Health summits 2012, 2013 and 2015
- International Symposia on Veterinary Epidemiology and Economics proceedings 1976-2012
- Global Elimination of Dog Mediated Rabies conference 2015
- Reports from WHO, OIE, FAO, GARC, HSI, IFAW, WSPA/WAP, ICAM, Gates Foundation, Dogs Trust International and any projects they have been involved with e.g. Mission Rabies – to be solicited
- Hand searching any other initiatives mentioned in published papers, conference proceedings, reports to find any other interventions
- Contacting authors, experts and organisations for any further reports (see appendix A)

**Search terms**

**Medline In-Process & Non-Indexed Citations and Ovid MEDLINE: 1946 - Present**

dog.mp. OR dogs.mp. OR canine.mp. OR canines.mp. OR canis.mp. OR exp Dogs/

AND

rabies.mp. OR rabid.mp. OR exp Rabies/ OR exp Rabies virus/

AND

vaccination.mp. OR vaccine.mp. OR vaccines.mp. OR vaccinate.mp. OR immunis$.mp. OR immuniz$.mp. OR exp Vaccines/ OR exp Rabies Vaccines/ OR exp Vaccination/ OR exp Immunization/ OR sterilis$.mp. OR steriliz$.mp. OR dog population management.mp. OR animal birth control.mp. OR neuter$.mp. OR spay$.mp. OR spey$.mp. OR castrat$.mp. OR ovariohysterectomy.mp. OR fertility control.mp. OR gonadectomy.mp. OR ABC.mp.

**CAB Abstracts (Ovid): 1910 – Present**

dog.mp. OR dogs.mp. OR canine.mp. OR canines.mp. OR canis.mp. OR exp Dogs/

AND

rabies.mp. OR rabid.mp. OR exp rabies/ OR exp Rabies virus/

AND

vaccination.mp. OR vaccine.mp. OR vaccines.mp. OR vaccinate.mp. OR immunis$.mp. OR immuniz$.mp. OR exp vaccines/ OR exp vaccination/ OR exp immunization/ OR

sterilis$.mp. OR steriliz$.mp. OR dog population management.mp. OR animal birth control.mp. OR neuter$.mp. OR spay$.mp. OR spey$.mp. OR castrat$.mp. OR ovariohysterectomy.mp. OR fertility control.mp. OR gonadectomy.mp. OR ABC.mp.

**Global Health (Ovid) 1910 to 2017 Week 19**

dog.mp. OR dogs.mp. OR canine.mp OR canines.mp. OR canis.mp. OR exp dogs/

AND

rabies.mp. OR rabid.mp. OR exp rabies/ OR exp Rabies virus/

AND

Vaccination.mp. OR vaccine.mp. OR vaccines.mp. OR vaccinate.mp. OR immunis$.mp. OR immuniz$.mp. OR exp vaccination/ OR exp immunization OR exp vaccines/ OR exp immunization programmes/ OR exp disease prevention/ OR

sterilis$.mp. OR steriliz$.mp. OR dog population management.mp. OR animal birth control.mp. OR neuter$.mp. OR spay$.mp. OR spey$.mp. OR castrat$.mp. OR ovariohysterectomy.mp. OR fertility control.mp. OR gonadectomy.mp. OR ABC.mp.

**Study selection**

**Inclusion and exclusion criteria**

| Criteria | Inclusion | Exclusion |
| --- | --- | --- |
| Population | Canine rabies control programs | Sterilisation program only or other DPM program only with no rabies vaccination performed |
| Intervention | Surgical sterilisation with rabies vaccination | Culling, other forms of fertility control |
| Comparator | Rabies vaccination only |  |
| Outcome | One or more of the following outcomes recorded - number of dog bites, number of confirmed or suspected rabid dog bites, number of PEP doses administered, number of dog rabies cases, number of human rabies cases, dog population turnover, change in health and welfare of dogs, changed public perception of dogs, changed public perception towards the intervention | None of the listed outcomes recorded |
| Language | All languages if translation available | Translation not available |
| Publication type | Published and grey literature | Unable to obtain full study details |

Discuss any papers queried for inclusion with supervisors

JS to assess random sample of 10% to check for concordance

**Quality assessment**

Appropriate risk of bias and quality assessment tools for study types that are included.

**Data extraction**

A form will be designed to collect data on study characteristics, intervention effort and intervention effectiveness. Relevant outcomes will be extracted measuring reduction of rabies risk and other related indicators.

- Location – country
- Setting – urban, peri-urban or rural
- Estimated dog population
- Numbers of dogs vaccinated and/or sterilised
- % coverage of dogs vaccinated and/or sterilised
- Implemented by international NGO/local NGO/ government/combination
- Length of intervention and whether carried out continually, fixed term, one-off, repeated annually
- Outcomes measured for success for reduction of rabies risk
- Number of suspected/confirmed rabid dog bites or PEP doses administered
- Number of dog rabies cases
- Number of human rabies cases
- Outcomes measured for success for related outcomes
- Dog population turnover
- Dog population size
- Dog population demographic changes
- Number of dog bites
- Health changes of free-roaming dogs
- Welfare changes of free-roaming dogs
- Human behaviour changes (KAP surveys or increase in cooperation with project or increase in people seeking vaccination/sterilisation for their dogs)

**Data synthesis**

PRISMA reporting guidelines

**Dissemination**

Publication in peer-reviewed journal and lay summary.

**References**

BERAN, G. W. & FRITH, M. 1988. Domestic Animal Rabies Control: An Overview. *Reviews of Infectious Diseases,* 10**,** S672-S677.

MORTERS, M. K., RESTIF, O., HAMPSON, K., CLEAVELAND, S., WOOD, J. L. & CONLAN, A. J. 2013. Evidence-based control of canine rabies: a critical review of population density reduction. *J Anim Ecol,* 82**,** 6-14.

REECE, J. F. & CHAWLA, S. K. 2006. Control of rabies in Jaipur, India, by the sterilisation and vaccination of neighbourhood dogs. *Veterinary Record,* 159**,** 379.

WORLD HEALTH ORGANISATION. WHO Expert Consultation on Rabies. Second Report (Vol.932). Geneva: *World Health Organization*. (2013).

**Appendix A: Letter for expert referral**

Dear x

I am a PhD student in the Centre for Evidence-based Veterinary Medicine at the University of Nottingham and am conducting a systematic review on the role of sterilisation in canine rabies control programs. The review will be part of a larger project aimed at developing evidence-based resources which we can make available to those working in the field of dog population management and/or rabies control.

The central question of the review is to what extent canine rabies control programs are affected by concurrent sterilisation initiatives. The review will compare the outcomes in programs which have performed sterilisation and vaccination with those that have performed vaccination only. The following outcomes will be examined – number of dog bites (including number of confirmed or suspected rabid dog bites), number of dog rabies cases or number of human rabies cases, human behaviour changes, dog demographic changes, such as population turnover and health and welfare changes.

The aim is to establish to identify trends, inconsistencies and gaps in the literature, and explore reasons for these. In addition to resources accessed by searching CAB Abstracts, Medline and Global Health databases, we are keen to identify any relevant grey literature – such as reports, presentations and publications describing such initiatives, to ensure our findings are as accurate and widely applicable as possible.

If you have any documents from projects which have measured any of the outcomes listed above, we would be very grateful if you would consider sharing them with us for inclusion in the review. Additionally, if you are aware of any other suitable projects, then please forward this email or contact me at: Abigail.Collinson@nottingham.ac.uk

The project has been ethically reviewed by the University of Nottingham panel. Please contact me if you would like further details or discussion.

Many thanks for your consideration,

Abi Collinson BVetMed MRCVS

PhD Student

Centre for Evidence-based Veterinary Medicine

School of Veterinary Medicine and Science

University of Nottingham

Sutton Bonington Campus

College Road

Loughborough

LE12 5RD

Email: Abigail.Collinson@nottingham.ac.uk

Supervisors:

Dr Rachel Dean BVMS PhD MSc DSAM(fel) MRCVS

Director of the Centre for Evidence-based Veterinary Medicine

University of Nottingham

Dr Jenny Stavisky BVM&S PhD MRCVS

Clinical Lecturer, Faculty of Medicine & Health Sciences

University of Nottingham

Prof Malcolm Bennett BVSc PhD MRCVS FRCPath

Professor of Zoonotic and Emerging Disease, Faculty of Medicine & Health Sciences

University of Nottingham
