# Supplementary material for: Evaluating the role of surgical sterilisation in canine rabies control: A systematic review of impact and outcomes
Source: PLoS Negl Trop Dis. 2020 Aug 26;14(8):e0008497. doi: 10.1371/journal.pntd.0008497 (PMC7449413; doi:10.1371/journal.pntd.0008497)
Supplement: S2 File — (DOCX) [file pntd.0008497.s002.docx]

# S2: Example data extraction form

| Title |  |
| --- | --- |
| First author |  |
| Year |  |
| Publication type |  |
| Study design |  |
| Country, region |  |
| Setting |  |
| Baseline scenario |  |
| Intervention (including methods e.g. fixed point, door to door..)  Free of charge? |  |
| Length of intervention (and study length) |  |
| Regularity |  |
| Implemented by |  |
| Aims/objectives of intervention/study/paper |  |
| Estimated dog population size of intervention area: |  |
| Method for estimating dog population size |  |
| Estimated human population and or human:dog density and method:  (dogs per human) |  |
| No. dogs vaccinated (coverage) |  |
| Method for estimating vaccination coverage |  |
| No. dogs sterilised (coverage) |  |
| Method for estimating sterilisation coverage |  |
| Outcomes measured |  |
| Methods for obtaining outcome measurements |  |
| Time after intervention that outcomes were measured |  |
| Results |  |
| Other successes reported |  |
| Challenges discussed: |  |
| Other issues that may have impacted program: |  |
| Reasons given for including sterilisation: |  |
