# Supplementary material for: Evaluating the role of surgical sterilisation in canine rabies control: A systematic review of impact and outcomes
Source: PLoS Negl Trop Dis. 2020 Aug 26;14(8):e0008497. doi: 10.1371/journal.pntd.0008497 (PMC7449413; doi:10.1371/journal.pntd.0008497)
Supplement: S3 File — (DOCX) [file pntd.0008497.s003.docx]

# S3: Modified AXIS checklist

|  | QUESTION | Y | N | Don’t know/Comment |
| --- | --- | --- | --- | --- |
|  | INTRODUCTION |  |  |  |
| 1 | Were the objectives of the study clear? |  |  |  |
|  | METHODS |  |  |  |
| 2 | Was the study design appropriate for the stated aim(s)? |  |  |  |
| 3 | Was the sample size justified |  |  | n/a |
| 4 | Was the target/reference population clearly defined (is it clear who the research was about) |  |  |  |
| 5 | Was the sample frame taken from an appropriate population base so that it closely represented the target/reference population under investigation |  |  |  |
| 6 | Was the selection process likely to select subjects representative of target/reference population under investigation? |  |  |  |
| 7 | Were measures undertaken to address and categorise non-responders? |  |  | n/a |
| 8 | Were the risk factor and outcome variables measured appropriate to the aim of the study? |  |  |  |
| 9 | Were the risk factor and outcome variables measured correctly using instruments/measurements that had been trialled, piloted or published previously? |  |  |  |
| 10 | Is it clear what was used to determine statistical significance |  |  |  |
| 11 | Were the methods (incl statistical methods) sufficiently described to enable them to be repeated? |  |  |  |
|  | RESULTS |  |  |  |
| 12 | Were the basic data adequately described? |  |  |  |
| 13 | Does the response rate raise concerns about non-response bias? |  |  | n/a |
| 14 | If appropriate, was info about nonresponders described? |  |  | n/a |
| 15 | Were the results internally consistent? |  |  |  |
| 16 | Were the results presented for all the analyses described in the methods |  |  |  |
|  | DISCUSSION |  |  |  |
| 17 | Were the author’s discussions and conclusions justified by the results? |  |  |  |
| 18 | Were limitations of the study discussed? |  |  |  |
|  | OTHER |  |  |  |
| 19 | Were there any funding sources or conflicts of interest that may affect the author interpretation of the results ? |  |  |  |
| 20 | Was ethical approval or consent of participants attained? |  |  |  |
